# BICP0 Negatively Regulates TRAF6-Mediated NF-κB and Interferon Activation by Promoting K48-Linked Polyubiquitination of TRAF6

**DOI:** 10.3389/fmicb.2019.03040

**Published:** 2020-01-08

**Authors:** Chong Cao, Ran An, YueYang Yu, HaiYue Dai, ZheHui Qu, MingChun Gao, JunWei Wang

**Affiliations:** Heilongjiang Key Laboratory for Animal Disease Control and Pharmaceutical Development, College of Veterinary Medicine, Northeast Agricultural University, Harbin, China

**Keywords:** BICP0, TRAF6, interferon, NF-κB, motif, ubiquitin

## Abstract

The infected cell protein 0 (BICP0) is an immediate early protein encoded by BHV-1, and its RING finger domain, which endows BICP0 with intrinsic E3 ubiquitin ligase activity, is common in all ICP0 proteins. Tumor necrosis factor receptor-associated factor 6 (TRAF6) is one of the TRAF family members and is ubiquitously expressed in mammalian tissues. TRAF6 forms the MyD88-TRAF6-IRF7 complex and activates interferon induction in the TLR (Toll-like receptors) and the RLR (RIG-I-like receptor) pathway. Previous studies showed that BICP0 reduced IFN-β promoter activity by interacting with IRF7. In this study, we found that BICP0 promoted the K48-ubiquitination and degradation of TRAF6 through the ubiquitin proteasome system. The interaction between BICP0 and TRAF6 is a prerequisite for ubiquitination modification, and the 346-PAERQY-351 of BICP0 is indispensable. The motif mutation experiments showed that the tyrosine 351 of BICP0 is the key amino acid involved. Further studies demonstrated that BICP0 suppressed the NF-κB pathway via the interference of TRAF6. Moreover, degradation of TRAF6 protein influenced the K63-linked ubiquitination of IRF7 and activation of interferon promoter. Collectively, these findings indicate that the BICP0 protein suppresses the inflammation signaling and IFN production by K48-linked polyubiquitination of TRAF6 and may further clarify the immune evasion function of BICP0.

## Introduction

Bovine herpesvirus 1 (BHV-1) is an enveloped virus belonging to the *alphaherpesvirus* subfamily, and is a significant bovine pathogen that leads to abortions, genital disorders, pneumonia, conjunctivitis, and “shipping fever,” which is an upper respiratory infection (Tikoo et al., 1995). Immunosuppression caused by BHV-1 infection triggers bovine respiratory disease complex (BRDC). As a poly-microbial disease caused by viral infection and stress, BRDC causes significant economic losses to the global cattle industry (Muylkens et al., 2007). The Infected Cell Protein 0 encoded by bovine herpesvirus-1 (BICP0) is important for the regulation of lytic and latent viral infections (Saira et al., 2008). Like the related proteins expressed by other *alphaherpesvirus* that infect mammalian species, BICP0 has a C3HC4 zinc RING finger domain in the amino-terminus, which is crucial for activating viral transcription and productive infection (Parkinson and Everett, 2000; Saira et al., 2008; Boutell and Everett, 2013). Aside from being one of the important virulence proteins of BHV-1, BICP0 also has an immunosuppressive function. The RING finger domain of BICP0 is essential for E3 ubiquitin ligase activity and leads to the ubiquitination and the subsequent degradation of a number of immune defense proteins. For example, BICP0 can directly catalyze IκBα ubiquitination (Diao et al., 2005). BICP0 also causes a decrease in IRF3 protein levels via the ubiquitin-dependent proteolysis pathway (Saira et al., 2007). PML-NB (promyelocytic leukemia protein-containing nuclear body) is a specific anti-viral organelle which regulates apoptosis and innate immune responses (Scherer and Stamminger, 2016). Many DNA viruses can recombine or split PML-NB, thereby increasing the copy number of the virus. Studies have shown that BICP0 co-localizes with and disrupts PML-NB (Parkinson and Everett, 2000; Inman et al., 2001). On the other hand, it was observed that BICP0 mediates the co-localization of IRF7 with nuclear structures that may be PML-NB in transfected cells, and that the interaction between BICP0 and IRF7 impairs activation of IFN-β promoter activity but does not change IRF7 protein levels (Saira et al., 2009). BICP0 thus reduces the ability of the IFN-β promoter in a manner correlated with IRF3 degradation, IRF7 interaction, and PML-NB dissolution, which has become a strategy used to destroy inherent innate antiviral defenses (Gaudreault and Jones, 2011).

Tumor necrosis factor receptor-associated factor 6 (TRAF6) is one of the TRAF family members, and is one of the most extensively investigated proteins in inflammatory responses (Lalani et al., 2018). TRAF6 is widespread in mammalian tissues and is conserved among species, and it consists of a RING finger domain in the N-terminal, followed by five Zn finger domains, and a C-terminal TRAF domain (containing a coiled-coil TRAF-N domain and a TRAF-C domain) (Cao et al., 1996; Ishida et al., 1996). The RING finger domain of TRAF6 possesses E3 ubiquitin ligase activity, which is essential for TRAF6 in the NF-κB activation downstream of TLRs (Toll-like receptors) (Akira and Takeda, 2004). TRAF6 forms an ubiquitin-binding enzyme complex with Ubc13 (Ubiquitin-conjugating enzyme 13) and Uev1A (ubiquitin-conjugating enzyme E2 variant 1) to promote the synthesis of lysine 63 (K63)–linked polyubiquitin chains (Deng et al., 2000). This K63-linked ubiquitination not only regulates protein functions and the interaction among proteins but also upregulates autophagic degradation. In general, K63-linked ubiquitination mediated by TRAF6 triggers signal transduction through the activation of downstream proteins (Sun and Chen, 2004). The protein kinase TAK1 (TGF beta-Activated Kinase 1) has been identified as one of the targets of TRAF6 and activated TAK1, which then triggers activation of canonical NF-κB by phosphorylating the IκB kinase complex (IKKα, IKKβ, and IKKγ) (Wang et al., 2001; Akira and Takeda, 2004). On the other hand, phosphorylation of MKK6 by TAK1 leads to activation of the JNK-p38 kinase pathway (Wang et al., 2001). TRAF6 also participates in autophagy stimulation by mediating Lys63-linked polyubiquitination of ULK1 (Nazio et al., 2013) and BECLIN-1 (Shi and Kehrl, 2010). Non-degradative ubiquitination by TRAF6 stimulates ULK1 self-association, which is a prerequisite for its kinase activity. Although TRAF6 has a well-established role in the regulation of both TAK1 and JNK signaling (Sakurai, 2012), the question of whether TRAF6 also controls autophagy through these kinases remains largely unexplored (Antonioli et al., 2017). In addition, TRAF6 may direct the activation of phosphoinositide 3-kinase (PI3K) when it binds to the TNFR superfamily, including TRANCE-R (also called RANK) and CD40, which regulate dendritic cell and osteoclast function (Wong et al., 1999; Arron et al., 2001). TRAF6 has also been shown to form the MyD88-TRAF6-IRF7 complex and activate interferon induction in the TLRs/IL-1 pathway (Honda et al., 2004; Kawai et al., 2004; Takaoka et al., 2005) and in the RLR (RIG-I-like receptor) pathway (Konno et al., 2009).

In this study, we demonstrated that BICP0 promotes the K48-linked ubiquitination of TRAF6, which then leads to the TRAF6 degradation by the ubiquitin proteasome system (UPS). The interaction between BICP0 and TRAF6 requires the involvement of a conservative motif, 346-PAERQY-351, of BICP0. By generating amino acid mutants, we found that the tyrosine 351 in the motif of BICP0 is the key amino acid involved. Further research showed that the degradation of TRAF6 mediated by BICP0 inhibited the functioning of TRAF6 on the NF-κB pathway. Moreover, the activation of the interferon pathway by IRF7 and TRAF6 is also affected by BICP0. Taken together, our study may provide new insights for the complex mechanism by which BICP0 regulates the innate antiviral immune response.

## Materials and Methods

### VSV, Baculovirus, and Plasmids

**FIGURE 1:**
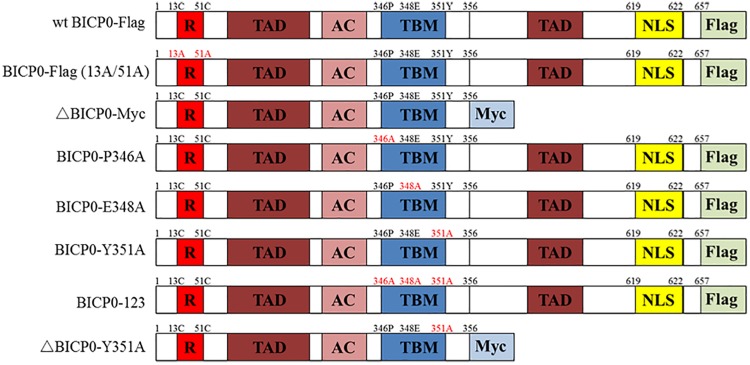
Schematic illustration of BICP0 and its mutants. R, zinc RING finger; TAD, transcriptional-activation domains; AC, acidic domain; TBM, TRAF6-binding motif; NLS, nuclear localization sequence; Flag, Flag-Tag sequence; Myc, Myc-Tag sequence.

The vesicular stomatitis virus (VSV) was stored in −80°C prior to use. The recombinant baculovirus (RE-BICP0-FLAG), which carries the Flag-tagged BICP0 gene, was constructed by our laboratory (data not shown). The baculovirus strain was propagated and titrated in insect cells determined by 50% tissue culture infective doses (TCID_50_) as described previously (Shao et al., 2015). The plasmid pcDNA3.1-BICP0-Flag expresses Flag-tagged wild type BICP0 (wt BICP0) under the control of the human cytomegalovirus (CMV) promoter. The mutant BICP0-Flag (13A/51A) contains site mutations within two conserved cysteine residues of the RING finger of Flag-tagged wt BICP0. The Myc-tagged N-terminal truncation mutants (ΔBICP0-Myc) were generated by standard PCR. To generate the motif mutants BICP0-P346A (pcDNA3.1-BICP0-Flag-P346A), BICP0-E348A (pcDNA3.1-BICP0-Flag-E348A), BICP0-Y351A (pcDNA3.1-BICP0-Flag-Y351A), ΔBICP0-Y351A (pcDNA3.1-ΔBICP0-Myc-Y351A), and BICP0-123 (pcDNA3.1-BICP0-Flag-123), alanine was substituted for the corresponding amino acids by PCR (Figure 1). Bovine IRF7 and TRAF6 genes were amplified from bovine cDNA and inserted into pcDNA3.1 (+) expression vectors; the recombinant plasmids are named pcDNA3.1-IRF7-HA, pcDNA3.1-TRAF6-HA, and pcDNA3.1-TRAF6-Flag, respectively. The plasmid pGL-3κB-luc (NF-κB-luc) was purchased form Promega. The IFN-β promoter construct (IFN-β-luc) was constructed by inserting the promoter region of IFN-β into the appropriate sites of the pGL3 vector.

### Cell Lines and Reagents

Madin Darby bovine kidney (MDBK) cells, HEK293T cells, and HeLa cells were maintained in Dulbecco’s Modified Eagle’s Medium (DMEM; Gibco) supplemented with 10% fetal bovine serum (FBS; Gibco) and penicillin (100 U/ml)-streptomycin (0.1 mg/ml) at 37°C in a 5% CO_2_ incubator. The antibodies used in this study were obtained from the following manufacturers: rabbit polyclonal antibodies to anti-Flag (GTX115043), anti-HA (GTX115044), anti-Myc (GTX115046), and anti-GAPDH (GTX100118) were purchased from GeneTex, Inc. (United States). Antibody against TRAF6 (A5724) and Anti-Flag magnetic beads (B26102) were purchased from Bimake. Anti-HA magnetic beads (HY-K0201), MG132, and chloroquine were purchased from MedChemExpress (MCE). The rabbit monoclonal antibodies against Ubiquitin (ab134953), K48-Ubiquitin (ab140601), and K63-Ubiquitin (ab179434) were purchased from Abcam. Horseradish peroxidase (HRP)-conjugated secondary antibody was purchased from ZSGB-BIO. The dual-luciferase reporter assay system was obtained from Promega. Sodium butyrate and Lipofectamine 2000 were purchased from Thermo Fisher Scientific, Inc.

### Transduction and Transfection

Madin Darby bovine kidney cells (∼2 × 10^6^) were seeded in 6-well plates 24 h before transduction with either the RE-BICP0-FLAG or an empty control baculovirus. The complete DMEM media contains 3 mM sodium butyrate, which enhances protein expression and transduction efficiency of the virus. After 24 h stimulation, MDBK cells were washed three times in ice-cold Tris-buffered saline (TBS), lysed, and subjected to western blot analysis as described below. For transfection, HEK293T cells (∼0.5 × 10^5^ and ∼1 × 10^7^) were seeded in 24-well plates and in 100 mm dishes, respectively. HeLa cells (∼2 × 10^6^) were seeded in glass-bottom dishes. Lipofectamine 2000 was used according to the manufacturer’s instructions.

### Western Blot Analysis and Immunoprecipitation

For western blot analysis, cells were lysed with RIPA buffer containing 50 mM Tris (pH 7.5), 150 mM NaCl, 1% NP-40, 0.25% sodium deoxycholate, 1 mM EDTA, 10 μg/ml aprotinin, 10 μg/ml leupeptin, and 1 mM PMSF. Lysates were incubated on ice for 30 min and clarified by centrifugation at 10,000 *g* at 4°C for 15 min. Protein concentrations were quantified using the BCA assay. For SDS-PAGE, proteins were mixed with 5 × sample loading buffer and boiled for 5 min. Proteins were separated in a 5–10% polyacrylamide gel and transferred onto a nitrocellulose membrane. Membranes were blocked at room temperature for 1 h in TBST (TBS-containing 0.05% Tween 20) that contained 5% milk. Membranes were then incubated overnight with the indicated primary antibody in TBST at 4°C. Afterward, the membrane was incubated in goat anti-rabbit immunoglobulin G (IgG)-HRP-conjugated secondary antibody for another 1 h at room temperature. At the end of each incubation, membranes were washed three times for 5 min each. Immunodetection was performed using enhanced chemiluminescence Western blotting detection reagents in accordance with the manufacturer’s protocol.

For immunoprecipitation assays, HEK293T cell lysates were incubated with Anti-HA/Anti-Flag magnetic beads for 2 h at room temperature according to the manufacturer’s instructions. After extensive washing, immunoprecipitated proteins were resolved using 5–10% SDS-PAGE and analyzed using western blotting using the indicated antibodies. Experiments were repeated at least three times and were observed to produce similar results.

### Confocal Imaging

The HeLa cells were transfected with the pcDNA3.1-BICP0-Flag, pcDNA3.1-BICP0-Flag-Y351A, or pcDNA3.1-TRAF6-HA (1 g each) for 36 h. Afterward, the cells were stained with the indicated antibodies and the images were acquired using a ZEISS confocal laser scanning system (ZEISS LSM800).

### Luciferase Assay

For the luciferase assay, HEK293T cells were transfected using the indicated plasmids. To normalize for transfection efficiency, 50 ng of pRL-TK *Renilla* luciferase plasmid was added to each transfection. At 36 h post-transfection, the HEK293T cells were harvested and whole cell extracts were prepared for the luciferase assay. Luciferase activity was measured using the Luciferase Assay System (Promega) with a GloMax^TM^ 20/20 Luminometer (Promega) and normalized relative to *Renilla* luciferase activities. Data were obtained from three independent transfections and are presented as the fold increase in luciferase activity (means ± SD) relative to the control.

### Statistical Analysis

Statistical analysis was performed using GraphPad Prism 5.0 (GraphPad Software, La Jolla, CA, United States^1^). Data are presented as mean ± SD. One-way analysis of variance (ANOVA) and Student’s *t*-tests were performed. *P*-values < 0.05 were considered statistically significant.

## Results

### BICP0 Reduces TRAF6 Protein Levels

**FIGURE 2:**
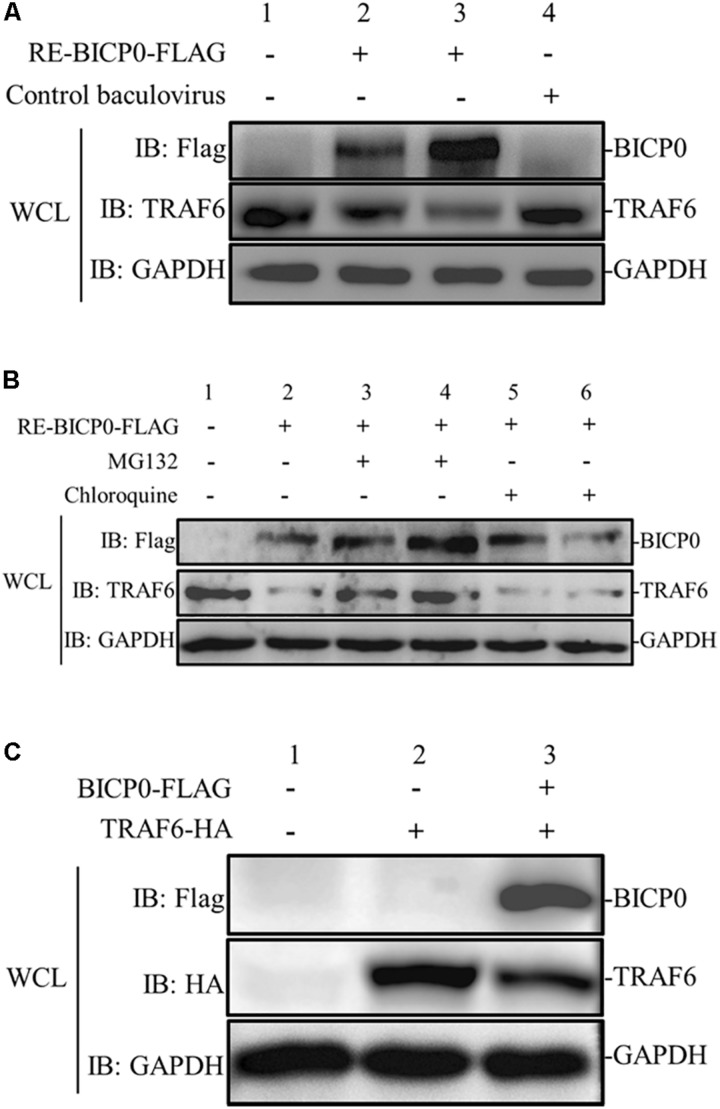
BICP0 leads to the degradation of TRAF6 protein through the proteasome. **(A)** BICP0 reduces TRAF6 protein levels in MDBK cells. The titer of RE-BICP0-FLAG and control baculovirus were 1 × 10^6^ TCID_50_/100 μl and 2 × 10^6^ TCID_50_/100 μl, respectively. MDBK cells were transduced with RE-BICP0-FLAG at 1 × 10^6^ TCID_50_ (lane 2) or 2 × 10^6^ TCID_50_ (lane 3), and the control baculovirus at 2 × 10^6^ TCID_50_ (lane 4). **(B)** TRAF6 was degraded by the 26S proteasome in MDBK cells. MDBK cells were transduced with 2 × 10^6^ TCID_50_ of RE-BICP0-FLAG for 20 h and treated with MG132 (lane 3: 1 μM and lane 4: 4 μM) or chloroquine (lane 5: 35 μM and lane 6: 70 μM) for another 4 h. MG132 reduced the degradation of TRAF6 in a dose-dependent manner. **(C)** BICP0 promoted TRAF6 degradation after transient transfection of HEK293T cells. HEK293T cells (∼0.5 × 10^5^) were co-transfected with pcDNA3.1-TRAF6-HA (0.5 μg each) and pcDNA3.1-BICP0-Flag (0.5 μg each). The control plasmid pcDNA3.1(+) (0.5 μg each) was used for balanced transfection efficiency. Densitometry analysis to quantify the ratio of TRAF6 to GAPDH is shown below. Experiments were repeated at least three times and produced similar results.

To determine the effect of BICP0 on innate immunity in the absence of other viral proteins, MDBK cells were transduced with different doses of RE-BICP0-FLAG, or with an empty control baculovirus. The results indicate that the presence of TRAF6 protein is significantly reduced in RE-BICP0-FLAG infected MDBK cells in a dose-dependent manner, and that there is no reduction in TRAF6 protein in the control baculovirus-infected MDBK cells (Figure 2A). It is known that the UPS and the autophagic lysosomal pathway (ALP) are the two major pathways for protein degradation; as such, we aimed to investigate if BICP0 causes the decrease of TRAF6 through the proteasome pathway or through lysosomal proteolysis. Consequently, we treated MDBK cells with the proteasome inhibitor MG132 or with the lysosome inhibitor chloroquine. The results showed that BICP0-induced TRAF6 degradation was rescued by MG132 but not by chloroquine, and that the decrease of TRAF6 was inhibited by MG132 in a dose-dependent manner (Figure 2B). Interestingly, the amount of BICP0 protein also increased with increasing dose of MG132 (Figure 2B). To test whether BICP0 can cause the decrease of TRAF6 protein in other cells, pcDNA3.1-BICP0-Flag and pcDNA3.1-TRAF6-HA were co-transfected into HEK293T cells (Figure 2C). Western blot results demonstrated that the overexpression of BICP0-Flag reduced TRAF6-HA protein expression in HEK293T cells, which was consistent with the conclusion derived from MDBK cells. Collectively, these data indicate that BICP0 can lead to the reduction of TRAF6 protein in MDBK cells and HEK293T cells, and TRAF6 was degraded through the proteasome pathway.

### BICP0 Promotes the K48-Linked Ubiquitination of TRAF6

**FIGURE 3:**
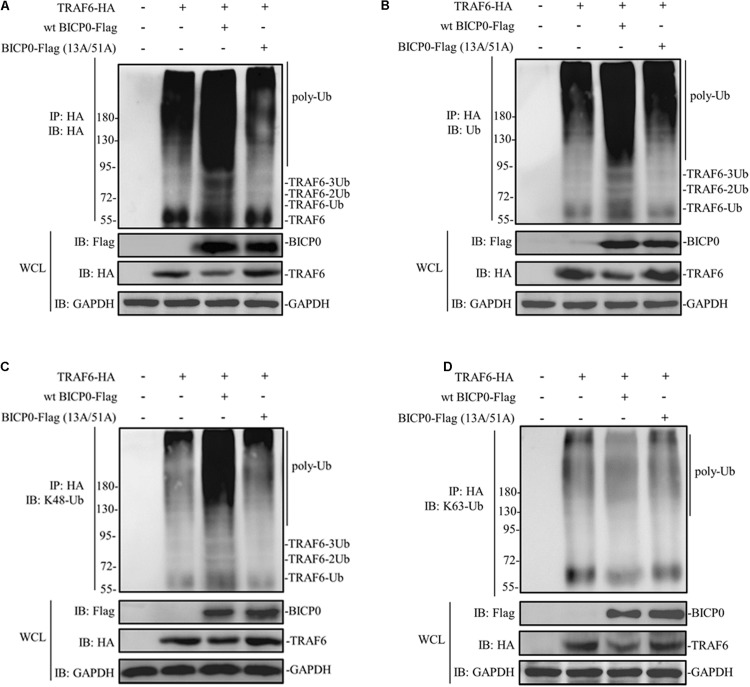
BICP0 promotes the K48-linked ubiquitination of TRAF6. At 36 h post-transfection, 10 μM MG132 was added for another 6 h, and cells were lysed on ice for 10 min. The supernatant was collected by centrifugation, which was immediately immunoprecipitated by anti-HA magnetic beads for 2 h at room temperature. Afterward, western blot was performed using antibodies against HA-tag/ubiquitin to detect TRAF6-HA **(A)** or ubiquitinated TRAF6 **(B–D)**. **(A)** TRAF6-HA protein bands appear diffuse in the presence of BICP0-Flag. **(B)** TRAF6-HA protein is ubiquitinated by BICP0-Flag. **(C,D)** The ubiquitin chains of BICP0-mediated TRAF6 ubiquitination are K48-linked. Experiments were repeated at least three times and produced similar results.

Considering that BICP0 is a RING-type E3 ubiquitin ligase, we hypothesized that TRAF6 degradation is dependent on the RING of BICP0. HEK293T cells were transfected with the indicated plasmids, and results were determined using immunoprecipitation and western blot assays. As can be seen in Figure 3A, TRAF6-HA protein was obtained by immunoprecipitation, and its band was detected using anti-HA antibody. Results of the co-transfected group showed that BICP0-Flag causes TRAF6-HA to separate into multiple bands, which were diffused and had a dark background. However, the band was significantly weaker in the TRAF6-HA transfection group and in the co-transfection group of TRAF6-HA with the BICP0-Flag (13A/51A) mutant. It is worth noting that only the wt BICP0-Flag can cause the decrease of TRAF6-HA in the whole cell lysate (WCL) samples. The antibody that specifically recognizes ubiquitin leads to similar results using anti-HA (Figure 3B). The results suggest that the ubiquitination of TRAF6-HA induced by BICP0-Flag depends on its E3 ligase activity. To confirm the type of ubiquitin chains, antibodies that specifically bind to K48-ubiquitin and K63-ubiquitin were used. The results showed that BICP0-Flag promotes the K48-linked ubiquitination of TRAF6-HA in transfected cells but not K63-linked ubiquitin chains (Figures 3C,D). These data suggest that the RING finger of BICP0 is important for its catalytic activity, which promotes the K48-ubiquitination of TRAF6 and leads to a subsequent reduction of TRAF6.

### BICP0 Interacts With TRAF6 and the Tyrosine 351 Is the Key Amino Acid

**FIGURE 4:**
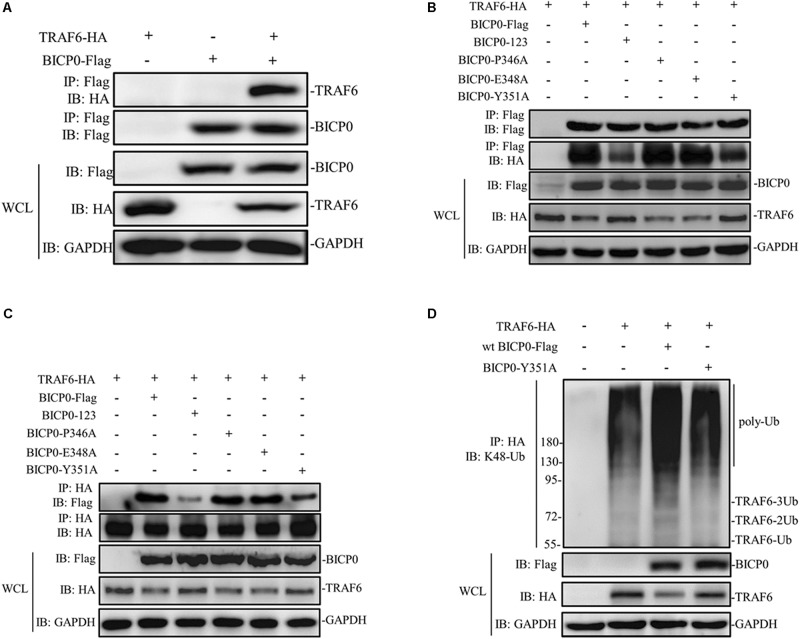
BICP0 binds TRAF6 though “346-PAERQY-351” and Y351 is the key residue. HEK293T cells (∼1 × 10^7^) were seeded in 100 mm dishes and transfected with the appropriate expression plasmids. At 36 h post-transfection, cell lysates were incubated with Anti-Flag/Anti-HA magnetic beads for 2 h at room temperature according to the manufacturer’s instructions. Western blot analysis with the indicated antibodies was performed. **(A)** Co-IP results by anti-Flag magnetic beads showed that BICP0 interacts with TRAF6 in the absence of other viral proteins. Co-IP **(B)** and reversed Co-IP **(C)** showed that the “346-PAERQY-351” motif of BICP0 responds for its interaction with TRAF6 and tyrosine 351 is the key amino acid. **(D)** Ubiquitination analyses revealed that the mutation of tyrosine 351 on BICP0 reduced TRAF6 K48-ubiquitination. Experiments were repeated at least three times and produced similar results.

**FIGURE 5:**
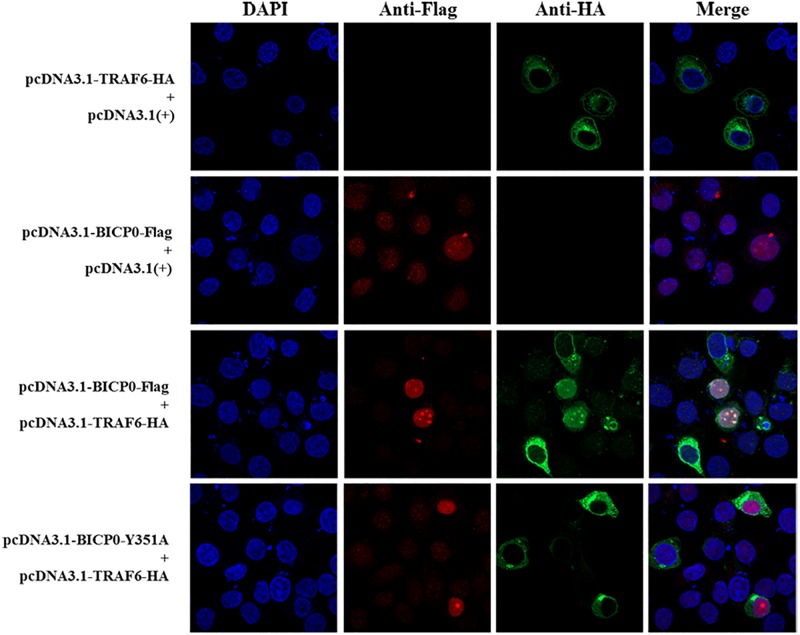
Confocal assay. The Hela cells were transfected with the pcDNA3.1-BICP0-Flag, pcDNA3.1-BICP0-Flag-Y351A, or pcDNA3.1-TRAF6-HA for 36 h. Afterward, the cells were stained with the indicated antibodies and subjected to confocal assay.

It is known that BICP0 mediates the ubiquitination and degradation of TRAF6; however, we aimed to investigate if the interaction between BICP0 and TRAF6 is necessary for ubiquitination modification, as it seems to have particular importance. To examine protein–protein interactions, Co-IP assay was carried out with the cell lysate prepared from HEK293T cells co-transfected with pcDNA3.1-BICP0-Flag and pcDNA3.1-TRAF6-HA. Immunoblot analysis using anti-HA antibodies revealed that BICP0 directly interacts with TRAF6 in the absence of other viral proteins (Figure 4A). In order to further study the domain structures involved in the BICP0-TRAF6 interaction, we analyzed the BICP0 protein sequence and found a conserved motif of BICP0—346-PAERQY-351. PCR was used to create several motif mutations: BICP0-123, BICP0-P346A, BICP0-E348A, and BICP0-Y351A. Co-IP and western blot assays showed that BICP0-123 has a significantly reduced ability to bind TRAF6 and that Y351 is the key amino acid responsible for the interaction between BICP0 and TRAF6 (Figure 4B). The result of reverse Co-IP is consistent with Co-IP (Figure 4C). To determine the role of the Y351 in the ubiquitination of TRAF6, HEK293T cells were co-transfected with pcDNA3.1-BICP0-Flag, or mutated pcDNA3.1-BICP0-Flag-Y351A and pcDNA3.1-TRAF6-HA. Ubiquitination analyses revealed that the mutation of Y351 on BICP0 reduced TRAF6 K48-ubiquitination (Figure 4D). To further identify the interaction between BICP0 and TRAF6, we also evaluated whether the BICP0 protein colocalizes with TRAF6 in HEK293T cells transfected with the indicated plasmids. The results showed the colocalization of BICP0 and TRAF6 in the nucleus; however, the BICP0-Y351A mutant lost its ability to colocalize with TRAF6 (Figure 5). It is worth noting that TRAF6 was introduced into the nucleus under increased expression of BICP0. Collectively, these data suggest that the direct binding of BICP0 with TRAF6 requires the involvement of a conservative peptide, and that Y351 is a key amino acid.

### BICP0 Negatively Regulates TRAF6-Mediated NF-κB and IFN-β Promoter Activation

**FIGURE 6:**
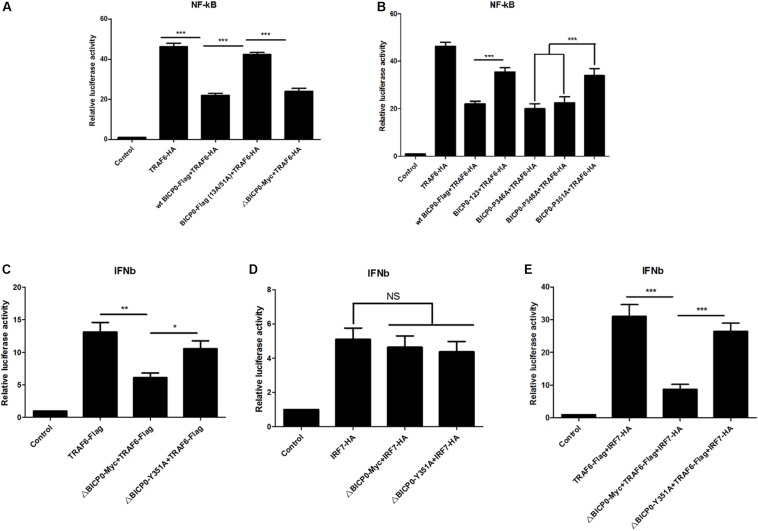
BICP0 inhibited the TRAF6-mediated activation of NF-κB and IFN-β promoter. HEK293T cells were transfected with NF-κB-luc (0.5 μg each) or IFN-β-luc (0.5 μg each) reporter plasmids in the presence of the indicated plasmids. *Renilla* luciferase plasmid (0.05 μg each) was used as an internal control. **(A,B)** At 36 h post-transfection, HEK293T cells were harvested and whole cell extracts were prepared for the luciferase assay. The RING domain **(A)** and Y351 **(B)** of BICP0 are both essential for disturbing the activation of TRAF6 on the NF-κB promoter. **(C,D)** At 24 h after transfection, HEK293T cells were infected with VSV (100 TCID_50_ per well) for 12 h before luciferase assays were performed. ΔBICP0-Myc inhibited TRAF6-mediated activation of IFN-β promoter **(C)** but had no effect on IRF7-HA **(D)**. TRAF6-Flag and IRF7-HA can synergistically activate the IFN-β promoter **(E)**, but the inhibition of ΔBICP0-Y351A was significantly weaker than ΔBICP0-Myc **(C,E)**. Data shown are presented as mean ± SD, *n* = 3. ^∗^*P* < 0.05, ^∗∗^*P* < 0.01, ^∗∗∗^*P* < 0.001. NS, not significant. Experiments were repeated at least three times and produced similar results.

Since BICP0 interacts with TRAF6, leading to its degradation by ubiquitination, we aimed to determine which signaling pathways downstream of TRAF6 become affected, as well as the types of changes that will occur. TRAF6 has been most studied in inflammation, so investigating its effect on the NF-κB pathway is a priority. To do this, luciferase tests were performed and results showed that overexpressed TRAF6-HA strongly activated the NF-κB promoter. The wt BICP0-Flag inhibited the activity of TRAF6-HA, but the inhibition effect of BICP0-Flag (13A/51A) was obviously weaker than the wt BICP0-Flag. Moreover, it was seen that the 357–657 aa at the carboxyl terminal of BICP0 is dispensable (Figure 6A). On the other hand, the BICP0-P346A and BICP0-E348A mutants exert the same inhibitory effect as the wt BICP0-Flag. However, the inhibitory effects of the BICP0-Y351A and BICP0-123 mutants were significantly weaker than wt BICP0 (Figure 6B). Results showed that the interaction between BICP0 and TRAF6 attenuated the relationship between TRAF6 and the NF-κB promoter, and that and the RING and Y351 of BICP0 are essential.

To explore the further impacts of BICP0 binding to TRAF6, we thought to examine the interferon pathways. It is known that IRF7 stimulates alpha/beta IFN (IFN-α/β) expression (Au et al., 1998; Marie et al., 1998) and functions as a significant regulator of the innate immune response. Another study found that TRAF6 binds and activates IRF7, which requires the ubiquitin ligase activity of TRAF6 (Kawai et al., 2004). Combining these findings with our experimental results, we speculated that BICP0 can inhibit the IFN-β pathway by interfering with TRAF6. To test this hypothesis, IFN-β-luciferase activity was measured. Earlier studies have shown that wt BICP0 inhibits the activation of IFN-β promoter by interacting with IRF7 in the nucleus, but a C-terminal deletion BICP0 mutant (4Δ*Nco*I, 1–607 aa) that lacks the nuclear localization signal (NLS) inhibited the IRF7-induced IFN-β promoter activity less efficiently than wt BICP0 (Saira et al., 2009). Therefore, in order to exclude the direct effect and interference of wt BICP0 on IRF7, tests were conducted with the ΔBICP0-Myc, which showed that TRAF6-Flag effectively activated the IFN-β promoter. Co-transfected ΔBICP0-Myc effectively inhibited the activity of TRAF6-Flag, while the inhibition of ΔBICP0-Y351A was significantly weaker than ΔBICP0-Myc (Figure 6C). On the other hand, IRF7-HA also activated the IFN-β promoter; however, co-transfected ΔBICP0-Myc or ΔBICP0-Y351A did not inhibit the activity of IRF7-HA (Figure 6D). To test whether TRAF6 degradation affected the activation of the IFN-β promoter by IRF7,HEK293T cells were co-transfected with pcDNA3.1-TRAF6-Flag or mutated pcDNA3.1-ΔBICP0-Myc-Y351A and pcDNA3.1-ΔBICP0-Myc and pcDNA3.1-IRF7-HA. Luciferase analyses revealed that TRAF6-Flag and IRF7-HA can synergistically activate the IFN-β promoter, whereas ΔBICP0-Myc effectively inhibited the co-activation; however, the inhibition of ΔBICP0-Y351A was significantly weaker than ΔBICP0-Myc (Figure 6E). In conclusion, BICP0 interacted with TRAF6 and promoted its degradation, and then inhibited TRAF6-activated IRF7. Most importantly, Y351 is the key amino acid involved in these interactions.

### BICP0 Weakens the Interaction Between TRAF6 and IRF7

**FIGURE 7:**
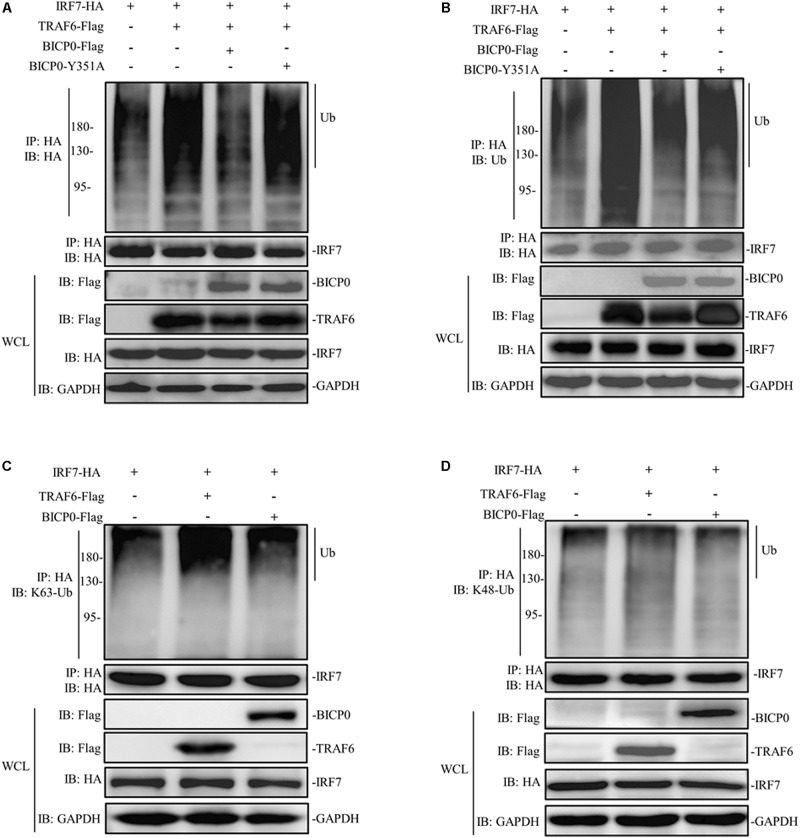
BICP0 eliminates the interaction between TRAF6 and IRF7. HEK293T cells (∼1 × 10^7^) were seeded in 100 mm dishes and transfected with the indicated plasmids (5 μg each). At 36 h post-transfection, cell lysates were incubated with anti-HA magnetic beads for 2 h at room temperature. Western blot analysis with the indicated antibodies was then performed. **(A,B)** BICP0 suppress IRF7 undergo ubiquitination. Co-immunoprecipitation samples were incubated with anti-HA antibody **(A)** and anti-ubiquitin antibody **(B)**. **(C,D)** TRAF6 mediates K63-linked ubiquitination of IRF7. Co-immunoprecipitation samples were incubated with antibodies against K63-ubiquitin **(C)** and K48-ubiquitin **(D)**.

The regulatory mechanism of IRF7 in the IFN pathway has been extensively studied. Like other transcriptional regulatory proteins, IRF7 also requires a series of post-translational modifications (PTMs); for example, in ubiquitination, sumoylation, acetylation, and phosphorylation are most important (Ling et al., 2019). It is worth noting that the activation of IRF7 requires ubiquitination, meanwhile, IRF7 will be ubiquitinated by TRAF6 at multiple sites both *in vitro* and *in vivo* (Ning et al., 2008). Given the interaction between IRF7 and TRAF6 and the effect of BICP0 on the stability of TRAF6 protein, we therefore speculated that BICP0 inhibits ubiquitination of IRF7 by affecting the TRAF6 protein level. To test this hypothesis, HEK293T cells were co-transfected with the indicated plasmids. Co-immunoprecipitation analyses showed that IRF7-HA was ubiquitinated by TRAF6-Flag, and ubiquitination of IRF7-HA was significantly decreased in the presence of BICP0-Flag. In contrast, BICP0-Y351A failed to interfere with the ubiquitination of IRF7-HA mediated by TRAF6-Flag (Figures 7A,B). Results showed that the interaction between BICP0 and TRAF6 can inhibit ubiquitination of IRF7 and that the tyrosine 351 in the conserved motif of BICP0 is the essential amino acid. We next sought to determine the ubiquitin chain type of IRF7, and the results showed that TRAF6-Flag promoted K63-linked ubiquitination of the IRF7-HA protein, whereas IRF7-HA was not modified by BICP0-Flag through ubiquitination (Figures 7C,D). Taken together, BICP0 interacts with TRAF6 and enhances the K48-linked ubiquitination and degradation of TRAF6, which subsequently leads to the decrease of K63-linked ubiquitination of IRF7.

## Discussion

Transient transfection tests are often used to study the function of individual proteins. As it is a protein encoded by bovine virus, the optimal experimental material to study the pathogenic mechanism of BICP0 is bovine cells. MDBK cells are commonly used as experimental materials for BHV-1 infection experiments; however, transfecting nucleic acids into MDBK is very difficult, and this low transfection efficiency (<5.0%) led to the failure in detecting any differences using the gene reporter technology assay (Osorio and Bionaz, 2017). The reasons for low transfection in MDBK cells is unclear. Alternatively, baculovirus mediates the high-efficiency transduction of nucleic acids into mammalian cells such as MDBK cells (Condreay et al., 1999). In this study, the use of baculovirus helped us to successfully deliver BICP0 proteins to MDBK cells; we then found that BICP0 reduced the expression of TRAF6 protein. As an effective foreign gene delivery system, baculovirus is a powerful tool that plays a more important role in the research of BHV-1 immune evasion. Co-transfection experiments showed that BICP0 reduced TRAF6 protein levels in transfected HEK293T cells. The results indicated that BICP0 can function in HEK293T cells; subsequent related studies were thus performed on HEK293T cells.

The use of proteasome inhibitor MG132 and lysosome inhibitor chloroquine demonstrated that a functional proteasome played a role in regulating TRAF6 protein levels, as seen in Figure 2B. These results were consistent with previous studies showing that BICP0 can cause protein degradation through the ubiquitin-proteasome pathway (Saira et al., 2007). The amount of BICP0 protein also increased with MG132 in a dose-independent manner, which seems to indicate that BICP0 is also degraded in the MDBK cells via the ubiquitin proteasome pathway. However, we do not know if the degradation of BICP0 is affected by self-ubiquitination or by other proteins in host cells. Further study is required and may lead to new discoveries regarding host antiviral mechanism.

Like other ICP0 proteins encoded by the *alphaherpesvirus* subfamily, the BICP0 of BHV-1 contains a RING finger near its N-terminal, and that its enzymatic activity is essential for its function (Henderson et al., 2005). From the above results, we have found that BICP0 can cause the decrease of TRAF6 through the ubiquitin proteasome pathway. It is known that ubiquitination is one of the most significant and most commonly existing protein PTMs in eukaryotes, which uses ubiquitin molecules to form different types of ubiquitin chains that lead to the modification of protein substrates (Kleiger and Mayor, 2014). Whether they go through the UPS or the ALP pathway, proteins undergo ubiquitination modification as a prerequisite. Therefore, we next studied the E3 ligase activity of BICP0. Ubiquitination analyses suggest that the RING finger of BICP0 is important for its catalytic activity, which promotes the K48-ubiquitination of TRAF6. On the other hand, TRAF6 is a non-conventional E3 ligase that promotes the synthesis of K63-ubiquitination. The K63 ubiquitin chain catalyzed by TRAF6 can not only modify other proteins but also modify itself. Modification of TAK1 leads to its activation (Wang et al., 2001; Akira and Takeda, 2004), and the modification of ULK1 (Nazio et al., 2013) and BECLIN-1 (Shi and Kehrl, 2010) leads to the activation of autophagy. However, no relevant studies have shown that the K63 ubiquitination modification of TRAF6 mediates its degradation. Regardless of whether K63 ubiquitination of TRAF6 worked, the results of this study confirmed that BICP0 modified TRAF6 by K48 ubiquitination and caused its degradation.

Protein ubiquitination involves the cooperation of three families of ubiquitin enzymes: E1, E2, and E3. Briefly, E1 activates ubiquitin with the help of ATP, which then binds ubiquitin for the formation of an E1-ubiquitin thiol ester linkage. Subsequently, ubiquitin is passed from E1 to E2. Finally, the E3 ubiquitin ligase binds to both the E2-ubiquitin complex and the protein substrate, promoting the transfer of ubiquitin onto the protein. Co-IP results showed that BICP0 interacted with TRAF6 without other viral proteins, and that the direct combination between BICP0 and TRAF6 guarantees ubiquitination modification. Apart from the RING finger, sequence analysis indicated BICP0 contains two transcriptional-activation domains (TADs), an acidic domain, and a consensus nuclear localization sequence (NLS; KRRR) (Figure 1) (Henderson et al., 2005). However, the mode of interaction of TRAF6 with receptors has been revealed by three available structures of complexes, including TRAF6–CD40 (Ye et al., 2002), TRAF6–TRANCE-R (Ye et al., 2002), and TRAF6–MAVS (Shi et al., 2015). The consensus P-X-E-X-X-Z sequence (x: any amino acid, Z: acidic or aromatic amino acid), which is also known as the TRAF6-binding motif, is in accordance with the receptor peptide residues of CD40, TRANCE-R, and MAVS directly interacting with TRAF6. Results showed that the TRAF6-binding motif is also in three IRAK adapter kinases (Ye et al., 2002) and in the intracellular domain of IFNλR1 (Xie et al., 2012). In this study, the most valuable finding in the sequence analysis of BICP0 is that the 346-PAERQY-351 peptide of BICP0 is conserved in different subtypes of BHV-1. Further analysis showed that 346-PAERQY-351 peptide is in accordance with TRAF6-binding motif. The proline 346 (P346), glutamic acid 348 (E348), and tyrosine 351 (Y351) of BICP0 are conserved according to the TRAF6-binding motifs. Mutation experiments showed that 346-PAERQY-351 of BICP0 is the binding domain of BICP0 and TRAF6 interaction, and that the aromatic amino acid (tyrosine 351) is the key interaction site. The residue in CD40 (F238) and TRANCE-R (Y349) is adjacent to several aromatic and basic residues of TRAF6, including R392, forming an amino-aromatic interaction (Park, 2018). This domain configuration of TRAF6 is the same as other mammalian TRAF family members, for example, TRAF2, TRAF3, and TRAF5 (Xie, 2013). TRAF2, TRAF3, and TRAF5 are able to interact with different overlapping motifs, such as P-X-Q-X-T (Chung et al., 2007; Hildebrand et al., 2010); however, the TRAF domain of TRAF6 binds specifically to the consensus TRAF6-binding motif, mainly through its TRAF-C domain (Ye et al., 2002). In this study, we did not test exactly which domain of TRAF6 mediated the interaction with BICP0, and more research needs to be done in the future.

Moreover, the carboxy terminus of BICP0 has a nuclear localization sequence, which can mediate the entry of BICP0 into the nucleus. Although TRAF6 has no nuclear localization sequence, co-immunoprecipitation indicates that BICP0 can bind to TRAF6. It is possible that TRAF6 can break through the nuclear membrane of the nucleus and enter the nucleus when there are enough of the BICP0 binds with TRAF6. On one hand, BICP0 causes K48 ubiquitination and degradation of TRAF6 in the cytoplasm; On the other hand, BICP0 may combines with TRAF6 and mediates its entry into the nucleus, thereby blocking TRAF6 from functioning in the cytoplasm. In subsequent studies, it will be necessary to explore the combined form of TRAF6 and BICP0.

TRAF6 is critical for the induction of many cytokines, such as inflammatory cytokines and interferons. In this study, we found that BICP0 directly binds to TRAF6 and affects its activation of NF-κB. ICP0 is the homolog of BICP0, and there is low similarity between BICP0 and ICP0 except in terms of the RING finger structure. Previous studies have shown that BICP0 and ICP0 could directly catalyze IκBα ubiquitination after transient transfection of HEK293T (Diao et al., 2005). Moreover, ICP0 had been shown to degrade various proteins such as p50/NF-κB1 (Zhang et al., 2013), MyD88, and Mal (also known as TIRAP) (van Lint et al., 2010). Therefore, we hypothesized that BICP0 can affect the function of other proteins in the NF-κB pathway through its E3 ligase. As an upstream molecule of TRAF6 in the NF-κB pathway, MyD88 recruits TRAF6 and forms a signal complex when the cell receives exogenous signal stimulation. We had found that BICP0 can also lead to the degradation of MyD88 (data unpublished); however, we do not know whether TRAF6 is involved in the binding between BICP0 and MyD88, and more work needs to be done.

The type I interferon (IFN-I)-inducing pathway is one of the most commonly stimulated signaling pathways during viral infection. Different pattern recognition receptors (PRRs) stimulated by exogenous stimulation will phosphorylate IRF3 and IRF7. Phosphorylated IRF3/7 then subsequently moves from the cytoplasm to the nucleus, and works together with activated NF-κB and ATF2/c-Jun to induce IFN-I production (Akira et al., 2006). Previous research has shown that TRAF6 also binds to IRF7 and results in IRF7 activation, and for this, the ubiquitin ligase activity of TRAF6 is required (Kawai et al., 2004; Ning et al., 2008). Furthermore, results show that MyD88-TRAF6-IRF7 complex regulates IFN-α production via TLR7, TLR8, and TLR9 (Honda et al., 2004; Kawai et al., 2004). In addition, TRAF6 mediates antiviral responses in RLR signaling that is triggered by viral DNA and RNA in the cytosol; this is different from TLR signaling and is important for the production of IFN-I and activation of NF-κB (Konno et al., 2009). As an immune-evasion gene encoded by BHV-1 that promotes productive infection, BICP0 reduces IFN-β promoter activity by causing the degradation of IRF3 in transient transfection studies (Henderson et al., 2005; Saira et al., 2007). BICP0 also impairs the activation of IFN-β promoter by interacting with the IRF7 protein, but it does not reduce IRF7 protein levels (Saira et al., 2007, 2009). However, it is not clear whether BICP0 interacts directly with IRF7 or with protein complexes containing IRF7 (Saira et al., 2009), and there have been no more developments in this field during the last decade. In this study, we showed that the interaction between BICP0 and TRAF6 promoted the degradation of TRAF6, which in turn caused the decrease of K63-linked ubiquitination of IRF7 and attenuated activation of the IFN-β promoter. Whether the binding of BICP0 and TRAF6 directly destroys the formation of MyD88-TRAF6-IRF7 complex is unknown, and follow-up work is currently being performed. In addition to the NF-κB and IFN pathway, TRAF6 may also direct the activation of mitogen-activated protein kinase (MAPK) (Wang et al., 2001), PI3K (Wong et al., 1999; Arron et al., 2001), and autophagy (Shi and Kehrl, 2010; Nazio et al., 2013). The effect of BICP0 on TRAF6 in these areas is worth investigating for future in-depth research.

In summary, this is the first study to demonstrate that BICP0 suppresses NF-κB signaling and IFN activation via TRAF6 interference. These results regarding BICP0 may help to further understand the interactions between viruses and hosts.

## Data Availability Statement

All datasets generated for this study are included in the article/supplementary material.

## Author Contributions

JW and CC designed the study and wrote the manuscript. RA, YY, and HD carried out the experiments. MG and ZQ analyzed the results. All authors read and approved the final manuscript.

## Conflict of Interest

The authors declare that the research was conducted in the absence of any commercial or financial relationships that could be construed as a potential conflict of interest.

## Footnotes

1 www.graphpad.com

## References

[B1] Akira S. Takeda K. (2004). Toll-like receptor signalling. *Nat. Rev. Immunol.* 4 499–511. 10.1038/nri1391 15229469

[B2] Akira S. Uematsu S. Takeuchi O. (2006). Pathogen recognition and innate immunity. *Cell* 124 783–801. 10.1016/j.cell.2006.02.015 16497588

[B3] Antonioli M. Di Rienzo M. Piacentini M. Fimia G. M. (2017). Emerging mechanisms in initiating and terminating autophagy. *Trends Biochem. Sci.* 42 28–41. 10.1016/j.tibs.2016.09.008 27765496

[B4] Arron J. R. Vologodskaia M. Wong B. R. Naramura M. Kim N. Gu H. (2001). A positive regulatory role for Cbl family proteins in tumor necrosis factor-related activation-induced cytokine (trance) and CD40L-mediated Akt activation. *J. Biol. Chem.* 276 30011–30017. 10.1074/jbc.M100414200 11406619

[B5] Au W. C. Moore P. A. LaFleur D. W. Tombal B. Pitha P. M. (1998). Characterization of the interferon regulatory factor-7 and its potential role in the transcription activation of interferon A genes. *J. Biol. Chem.* 273 29210–29217. 10.1074/jbc.273.44.29210 9786932

[B6] Boutell C. Everett R. D. (2013). Regulation of alphaherpesvirus infections by the ICP0 family of proteins. *J. Gen. Virol.* 94(Pt 3), 465–481. 10.1099/vir.0.048900-0 23239572

[B7] Cao Z. Xiong J. Takeuchi M. Kurama T. Goeddel D. V. (1996). TRAF6 is a signal transducer for interleukin-1. *Nature* 383 443–446. 10.1038/383443a0 8837778

[B8] Chung J. Y. Lu M. Yin Q. Wu H. (2007). Structural revelations of TRAF2 function in TNF receptor signaling pathway. *Adv. Exp. Med. Biol.* 597 93–113. 10.1007/978-0-387-70630-6-8 17633020

[B9] Condreay J. P. Witherspoon S. M. Clay W. C. Kost T. A. (1999). Transient and stable gene expression in mammalian cells transduced with a recombinant baculovirus vector. *Proc. Natl. Acad. Sci. U.S.A.* 96 127–132. 10.1073/pnas.96.1.127 9874783 PMC15104

[B10] Deng L. Wang C. Spencer E. Yang L. Braun A. You J. (2000). Activation of the IkB kinase complex by TRAF6 requires a dimeric ubiquitin-conjugating enzyme complex and a unique polyubiquitin chain. *Cell* 103 351–361. 10.1016/s0092-8674(00)00126-4 11057907

[B11] Diao L. Zhang B. Fan J. Gao X. Sun S. Yang K. (2005). Herpes virus proteins ICP0 and BICP0 can activate NF- κB by catalyzing IκBα ubiquitination. *Cell. Signal.* 17 217–229. 10.1016/j.cellsig.2004.07.003 15494213

[B12] Gaudreault N. Jones C. (2011). Regulation of promyelocytic leukemia (PML) protein levels and cell morphology by bovine herpesvirus 1 infected cell protein 0 (bICP0) and mutant bICP0 proteins that do not localize to the nucleus. *Virus Res.* 156 17–24. 10.1016/j.virusres.2010.12.010 21215282

[B13] Henderson G. Zhang Y. Jones C. (2005). The Bovine herpesvirus 1 gene encoding infected cell protein 0 (bICP0) can inhibit interferon-dependent transcription in the absence of other viral genes. *J. Gen. Virol.* 86(Pt 10), 2697–2702. 10.1099/vir.0.81109-0 16186222

[B14] Hildebrand J. M. Luo Z. Manske M. K. Price-Troska T. Ziesmer S. C. Lin W. (2010). A BAFF-R mutation associated with non-Hodgkin lymphoma alters TRAF recruitment and reveals new insights into BAFF-R signaling. *J. Exp. Med.* 207 2569–2579. 10.1084/jem.20100857 21041452 PMC2989778

[B15] Honda K. Yanai H. Mizutani T. Negishi H. Shimada N. Suzuki N. (2004). Role of a transductional-transcriptional processor complex involving MyD88 and IRF-7 in Toll-like receptor signaling. *Proc. Natl. Acad. Sci. U.S.A.* 101 15416–15421. 10.1073/pnas.0406933101 15492225 PMC523464

[B16] Inman M. Zhang Y. Geiser V. Jones C. (2001). The zinc ring finger in the bICP0 protein encoded by bovine herpesvirus-1 mediates toxicity and activates productive infection. *J. Gen. Virol.* 82(Pt 3), 483–492. 10.1099/0022-1317-82-3-483 11172088

[B17] Ishida T. Mizushima S. Azuma S. Kobayashi N. Tojo T. Suzuki K. (1996). Identification of TRAF6, a novel tumor necrosis factor receptor-associated factor protein that mediates signaling from an amino-terminal domain of the CD40 cytoplasmic region. *J. Biol. Chem.* 271 28745–28748. 10.1074/jbc.271.46.28745 8910514

[B18] Kawai T. Sato S. Ishii K. J. Coban C. Hemmi H. Yamamoto M. (2004). Interferon-α induction through Toll-like receptors involves a direct interaction of IRF7 with MyD88 and TRAF6. *Nat. Immunol.* 5 1061–1068. 10.1038/ni1118 15361868

[B19] Kleiger G. Mayor T. (2014). Perilous journey: a tour of the ubiquitin-proteasome system. *Trends Cell Biol.* 24 352–359. 10.1016/j.tcb.2013.12.003 24457024 PMC4037451

[B20] Konno H. Yamamoto T. Yamazaki K. Gohda J. Akiyama T. Semba K. (2009). TRAF6 establishes innate immune responses by activating NF-kB and IRF7 upon sensing cytosolic viral RNA and DNA. *PLoS One* 4:e5674. 10.1371/journal.pone.0005674 19479062 PMC2682567

[B21] Lalani A. I. Zhu S. Gokhale S. Jin J. Xie P. (2018). TRAF molecules in inflammation and inflammatory diseases. *Curr. Pharmacol. Rep.* 4 64–90. 10.1007/s40495-017-0117-y 29527458 PMC5839642

[B22] Ling T. Weng G. X. Li J. Li C. Wang W. Cao L. (2019). TARBP2 inhibits IRF7 activation by suppressing TRAF6-mediated K63-linked ubiquitination of IRF7. *Mol. Immunol.* 109 116–125. 10.1016/j.molimm.2019.02.019 30927622

[B23] Marie I. Durbin J. E. Levy D. E. (1998). Differential viral induction of distinct interferon-alpha genes by positive feedback through interferon regulatory factor-7. *EMBO J.* 17 6660–6669. 10.1093/emboj/17.22.6660 9822609 PMC1171011

[B24] Muylkens B. Thiry J. Kirten P. Schynts F. Thiry E. (2007). Bovine herpesvirus 1 infection and infectious bovine rhinotracheitis. *Vet. Res.* 38 181–209. 10.1051/vetres:2006059 17257569

[B25] Nazio F. Strappazzon F. Antonioli M. Bielli P. Cianfanelli V. Bordi M. (2013). MTOR inhibits autophagy by controlling ULK1 ubiquitylation, self-association and function through AMBRA1 and TRAF6. *Nat. Cell Biol.* 15 406–416. 10.1038/ncb2708 23524951

[B26] Ning S. Campos A. D. Darnay B. G. Bentz G. L. Pagano J. S. (2008). TRAF6 and the three C-terminal lysine sites on IRF7 are required for its ubiquitination-mediated activation by the tumor necrosis factor receptor family member latent membrane protein 1. *Mol. Cell. Biol.* 28 6536–6546. 10.1128/MCB.00785-08 18710948 PMC2577435

[B27] Osorio J. S. Bionaz M. (2017). Plasmid transfection in bovine cells: optimization using a realtime monitoring of green fluorescent protein and effect on gene reporter assay. *Gene* 626 200–208. 10.1016/j.gene.2017.05.025 28501631

[B28] Park H. H. (2018). Structure of TRAF Family: current understanding of receptor recognition. *Front. Immunol.* 9:1999. 10.3389/fimmu.2018.01999 30214450 PMC6125299

[B29] Parkinson J. Everett R. D. (2000). Alphaherpesvirus proteins related to herpes simplex virus type 1 ICP0 affect cellular structures and proteins. *J. Virol.* 74 10006–10017. 10.1128/jvi.74.21.10006-10017.2000 11024129 PMC102039

[B30] Saira K. Chowdhury S. Gaudreault N. da Silva L. Henderson G. Doster A. (2008). The zinc RING finger of bovine herpesvirus 1-encoded bICP0 protein is crucial for viral replication and virulence. *J. Virol.* 82 12060–12068. 10.1128/JVI.01348-08 18842710 PMC2593318

[B31] Saira K. Zhou Y. Jones C. (2007). The infected cell protein 0 encoded by bovine herpesvirus 1 (bICP0) induces degradation of interferon response factor 3 and, consequently, inhibits beta interferon promoter activity. *J. Virol.* 81 3077–3086. 10.1128/jvi.02064-06 17215277 PMC1866033

[B32] Saira K. Zhou Y. Jones C. (2009). The infected cell protein 0 encoded by bovine herpesvirus 1 (bICP0) associates with interferon regulatory factor 7 and consequently inhibits beta interferon promoter activity. *J. Virol.* 83 3977–3981. 10.1128/JVI.02400-08 19176627 PMC2663255

[B33] Sakurai H. (2012). Targeting of TAK1 in inflammatory disorders and cancer. *Trends Pharmacol. Sci.* 33 522–530. 10.1016/j.tips.2012.06.007 22795313

[B34] Scherer M. Stamminger T. (2016). Emerging role of PML nuclear bodies in innate immune signaling. *J. Virol.* 90 5850–5854. 10.1128/JVI.01979-15 27053550 PMC4907236

[B35] Shao J. Cao C. Bao J. Gao M. Wang J. (2015). Characterization of the biological activities and physicochemical characteristics of recombinant bovine interferon-α(1)(4). *Mol. Immunol.* 64 163–169. 10.1016/j.molimm.2014.11.011 25480392

[B36] Shi C. S. Kehrl J. H. (2010). TRAF6 and A20 regulate lysine 63-linked ubiquitination of Beclin-1 to control TLR4-induced autophagy. *Sci. Signal.* 3:ra42. 10.1126/scisignal.2000751 20501938 PMC6335036

[B37] Shi Z. Zhang Z. Zhang Z. Wang Y. Li C. Wang X. (2015). Structural insights into mitochondrial antiviral signaling protein (MAVS)-tumor necrosis factor receptor-associated factor 6 (TRAF6) signaling. *J. Biol. Chem.* 290 26811–26820. 10.1074/jbc.M115.666578 26385923 PMC4646334

[B38] Sun L. Chen Z. J. (2004). The novel functions of ubiquitination in signaling. *Curr. Opin. Cell Biol.* 16 119–126. 10.1016/j.ceb.2004.02.005 15196553

[B39] Takaoka A. Yanai H. Kondo S. Duncan G. Negishi H. Mizutani T. (2005). Integral role of IRF-5 in the gene induction programme activated by Toll-like receptors. *Nature* 434 243–249. 10.1038/nature03308 15665823

[B40] Tikoo S. K. Campos M. Babiuk L. A. (1995). Bovine herpesvirus 1 (BHV-1): biology, pathogenesis, and control. *Adv. Virus Res.* 45 191–223. 10.1016/s0065-3527(08)60061-5 7793325

[B41] van Lint A. L. Murawski M. R. Goodbody R. E. Severa M. Fitzgerald K. A. Finberg R. W. (2010). Herpes simplex virus immediate-early ICP0 protein inhibits Toll-like receptor 2-dependent inflammatory responses and NF-kB signaling. *J. Virol.* 84 10802–10811. 10.1128/JVI.00063-10 20686034 PMC2950559

[B42] Wang C. Deng L. Hong M. Akkaraju G. R. Inoue J. Chen Z. J. (2001). TAK1 is a ubiquitin-dependent kinase of MKK and IKK. *Nature* 412 346–351. 10.1038/35085597 11460167

[B43] Wong B. R. Besser D. Kim N. Arron J. R. Vologodskaia M. Hanafusa H. (1999). TRANCE, a TNF family member, activates Akt/PKB through a signaling complex involving TRAF6 and c-Src. *Mol. Cell* 4 1041–1049. 10.1016/s1097-2765(00)80232-4 10635328

[B44] Xie P. (2013). TRAF molecules in cell signaling and in human diseases. *J. Mol. Signal.* 8 7: 10.1186/1750-2187-8-7 23758787 PMC3697994

[B45] Xie Y. F. Cui Y. B. Hui X. W. Wang L. Ma X. L. Chen H. (2012). Interaction of IFNlambdaR1 with TRAF6 regulates NF-kB activation and IFNlambdaR1 stability. *J. Cell. Biochem.* 113 3371–3379. 10.1002/jcb.24213 22644879

[B46] Ye H. Arron J. R. Lamothe B. Cirilli M. Kobayashi T. Shevde N. K. (2002). Distinct molecular mechanism for initiating TRAF6 signalling. *Nature* 418 443–447. 10.1038/nature00888 12140561

[B47] Zhang J. Wang K. Wang S. Zheng C. (2013). Herpes simplex virus 1 E3 ubiquitin ligase ICP0 protein inhibits tumor necrosis factor alpha-induced NF-kB activation by interacting with p65/RelA and p50/NF-kB1. *J. Virol.* 87 12935–12948. 10.1128/JVI.01952-13 24067962 PMC3838126

